# Decoding glycans: deciphering the sugary secrets to be coherent on the implication

**DOI:** 10.1039/d0ra04471g

**Published:** 2020-09-15

**Authors:** Shreya Sharma, Shashank Shekhar, Bhasha Sharma, Purnima Jain

**Affiliations:** a Department of Chemistry, Netaji Subhas University of Technology Dwarka Sec-2 Delhi India sharmabhasha@gmail.com

## Abstract

Neoteric techniques, skills, and methodological advances in glycobiology and glycochemistry have been instrumental in pertinent discoveries to pave way for a new era in biomedical sciences. Glycans are sugar-based polymers that coat cells and decorate majority of proteins, forming glycoproteins. They are also found deposited in extracellular spaces between cells, attached to soluble signaling molecules, and are key players in several biological processes including regulation of immune responses and cell–cell interactions. Laboratory manipulations of protein, DNA and other macromolecules celebrate the accelerated research in respective fields, but the same seems unlikely for the complex sugar polymers. The structural complex polymers are neither synthesized using a known template nor are dynamically stable with respect to a cell's metabolic rate. What is more, sugar isomers—structurally distinct molecules with the same chemical formula—can be employed to construct varied glycans, but are almost impossible to tell apart based on molecular weight alone. The apparent lack of a glycan alphabet further reflects on an enduring question: how little do we know about the sugars? Evidently, glycan-based therapeutic potentials and glycomimetics are propitious advances for the future that have not been well exploited, and with a few conspicuous anomalies. Here, we contour the most notable contributions to enhance our ability to utilize the complex glycans as therapeutics. Diagnostic strategies concerning recurrent diseases and headways to address the challenges are also discussed.

## Abstract 2

A glycan toolbox for pathogenic and cancerous interventions. The review article sheds light on the sweet secrets of this complex structure.

## Graphical abstract

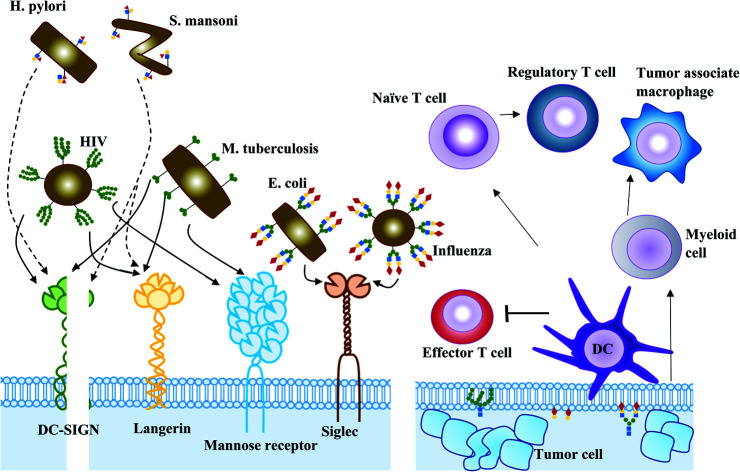

The decisive contribution of carbohydrates in proliferation of cells, signaling, structure, and morphology, alongside several diseases, has incentivized multitudinous endeavors to provide glycan-based remedies. Glycans are chains of monosaccharide units, varying in length from a few sugars to several hundreds. The astonishingly complex carbohydrate polymers form the most abundant family of organic molecules on the planet.^1,2^ Glycans are a vital class of biological molecules that compasses spectra of functions including growth, development, maintenance, and survival of an organism. They are key players in nearly every domain of physiology and etiology of almost every disease. Glycans coat the outermost surfaces of cells and secreted macromolecules, found in extracellular compartments and are prodigiously variegated. Differences in monosaccharide constituents, bonding between different monosaccharide units, anomeric states, branched structures, and several substitutions in linkage to the aglycon part are responsible for the diversification.^3^ Complex glycans are statutory for protein folds in polypeptide chains synthesized afresh, and influence the stability, solubility and trafficking of the terminal glycoprotein. About 1% of the human genome is devoted to the enzyme-catalyzed synthesis and allocation of glycans, and most of human proteins and lipids are believed to be post-translationally remoulded by glycans by a process called glycosylation.^4–6^ Major types of glycosylation seen in mammals are illustrated in Fig. 1. Inadequacy in glycosylation in humans and its relation to several diseases trace back to their role in the storage of tremendous amount of biological data. Glycosylation in proteins encompasses the inclusion of *O*-linked glycans, *N*-linked glycans, mucopolysaccharides, phosphorylated glycans, glycosylphosphatidylinositol anchors to peptide backbones as well as tryptophan residues undergoing C-mannosylation. Glycolipids are molecules composed of a glycan linked to a lipid ceramide; this type of glycoconjugate includes glycosphingolipids.

**Fig. 1:**
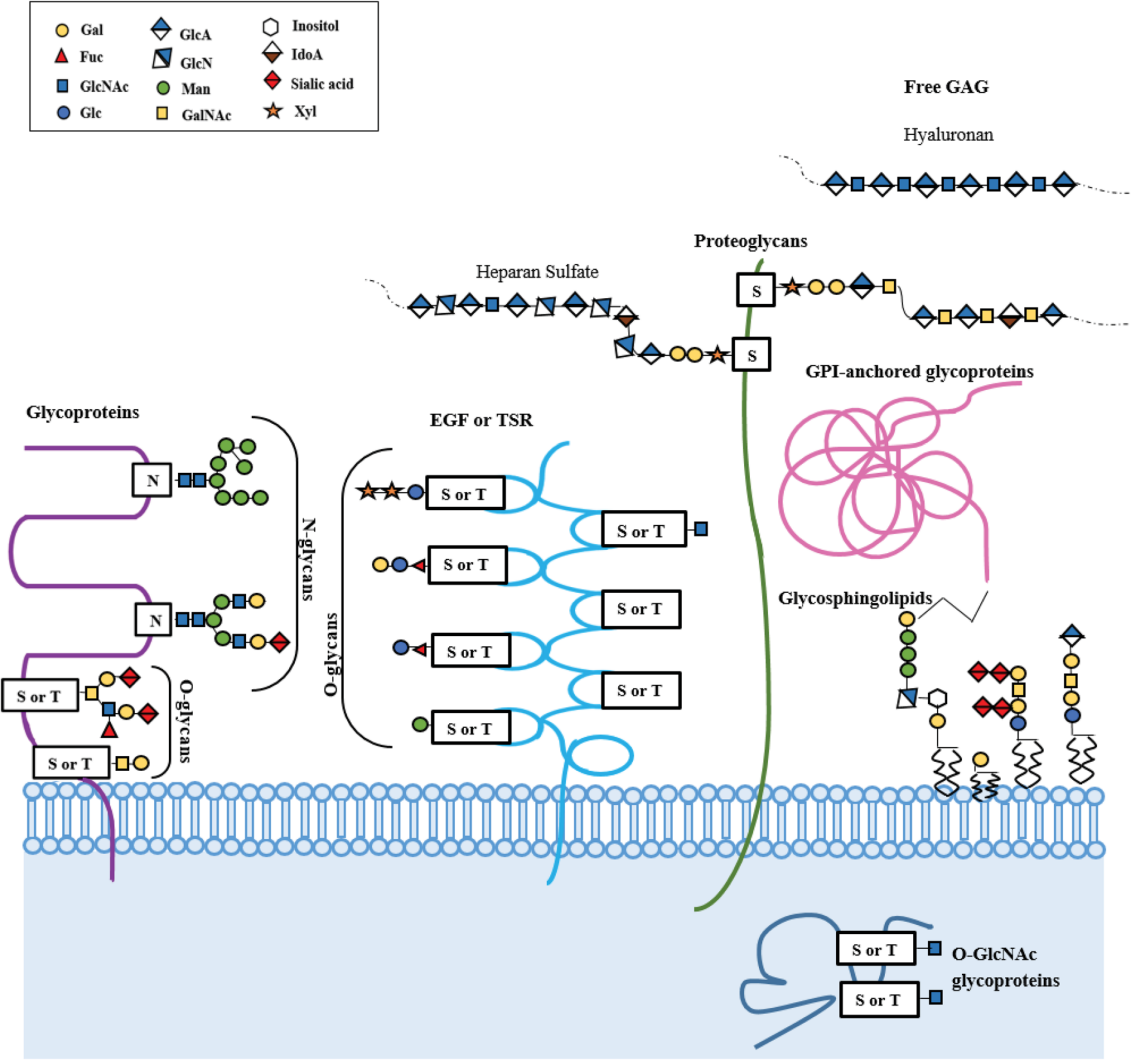
Major types of glycosylation and glycans in mammals. Glycans on mammalian cell surfaces are composed of 10 kinds of monosaccharides: glucose (Glc), galactose (Gal), mannose (Man), fucose (Fuc), *N*-acetylglucosamine (GlcNAc), *N*-acetylgalactosamine (GalNAc), xylose (Xyl), glucuronic acid (GlcA), iduronic acid (IdA), and sialic acid (Sia), which are connected *via* glycosyltransferase (GT)-catalyzed substitution from nucleotide pyrophosphate.

The biological role of glycans is categorized into: (1) structural and modulatory functions involving storage of nutrients and sequestration; (2) explicit identification by other molecules, specifically glycan-binding proteins; and (3) molecular mimicry of host glycans. Glycan-binding proteins are classified into intrinsic glycan-binding proteins and extrinsic glycan-binding proteins. The intrinsic functionalities are involved in direct cellular interactions, that is, recognition of extracellular glycan molecules from the same organism. The extrinsic functionalities are involved in the recognition of glycans from another organism, which constitute microbial adhesins, agglutinins, or toxins, and few arbitrate to form symbiotic relationships or host defense.^7,8^ This has empowered more prominent proficiency in the distinguishing proof, synthesis and assessment of peculiar glycan epitopes present on a surfeit of pathogens and cancerous cells. Glycans and glycan-binding proteins on the host cell are utilized by microbes to attach and invade the host cells, as carbon sources and toxins. Moreover, host glycans can be modified by the bacterial glycosyltransferases and glycosidases as a major share of the pathogenic procedure.^9^ Glycan coatings on the envelope of viruses are eminent for the evasion of the immunological surveillance of the host.^10,11^

The comprehensive grasp on genotype, which, in turn, controls the phenotype, was plausible with the discovery of structure–activity relationships of nucleic acids and proteins. Thereafter, the biological and biochemical knowledge was successfully translated into drug discovery endeavors. Even though the changes in the expression of glycans concerning cell conditions have been broadly acknowledged, progression in the sub-atomic premise of glycan functions has been fairly moderate compared with similar investigations of proteins and nucleic acids.^12^ This slow progress is partly due to the fact that the biosynthesis of glycans, unlike other biopolymers, is not template-driven and that the molecular basis governing glycan functions is not yet fully understood, presumably as a denouement of the huge structural complexity and heterogeneity of glycans. Howbeit, cloning of enzymes required for the synthesis of glycans, as well as elaborating the mechanism of action and substrate specificity, has now helped in the high-throughput analysis of a range of complex glycan structures. Glycomics have acquired appreciable heed. Innumerable genetic knock-out experiments have further enhanced our knowledge by scrutinizing at the organism level, the impingement on biosynthesis and/or catabolism of complex glycans. The loss-of-function studies utilizing gene knock-outs have bestowed significant structure-work associates for both complexes and linear polysaccharide units.^2,13^ Analysis of structural properties of individual glycans, distribution on cells or tissues and relationship with each other, and interactions with proteins and lipids foster a large volume of information, and result high-performance computing, overarching information, and data extraction. Several academicians and commercial organizations are involved in the integration of diversified data by utilizing various tools and instruments to develop novel searchable bioinformatics platforms. Some examples of the databases reported in the past few years are Glycostore,^14^ Glyco3D,^15^ GlyMDB,^16^ GLAD,^17^ UniLectin web platform,^18^ Carbohydrate structure database,^19^ UniCorn,^20^ UniCarbKB,^21^ and Kegg glycan.^22^ Computational data will aid in the progression of glycoanalytics to high-throughput utilization and quicker determination of new drug–disease relationships.

## Glycosylation: sweet or bitter for human pathogens?

### Bacterial pathogens

Carbohydrates featuring in bacterial glycans vary from trioses to dodecoses. Hexoses amid the spectrum of carbohydrates are the most diverse forms with respect to the variety in substitution patterns. Additionally, alterations by inclusion of acyl, alkyl, aminoacyl, phosphoryl, nucleoside groups, *etc.*, decorate the carbohydrates. Gram-positive bacteria contain peptidoglycans, lipopolysaccharides, lipooligosaccharides, capsular extracellular polysaccharides, capsular polysaccharides, and *N*-linked and *O*-linked glycoproteins. However, lipooligosaccharides and lipopolysaccharides are absent in Gram-positive bacteria. Gram-positive strains mark the presence of glycosylated lipo teichoic acids and wall teichoic acids as substitutes. Gram-positive mycobacteria manifest extra glycan varieties embracing arabinogalactan and lipoarabinomannan conjugates that highlight numerous furanose rings.^23^ Several surface lectins behave as adhesins that allow bacteria to attach to host cells, commence colonization, and establish cell and tissue tropism. For instance, *Escherichia coli* responsible for a spectra of diseases from enteritis to urinary tract infections and meningitis utilize several kinds of pili for adherence to the glycosylated surfaces of the host. *Haemophilus influenza* responsible for the respiratory diseases including otitis media and chronic bronchitis leading to chronic obstructive pulmonary disease utilizes a heavily weighted adhesion HMW1 glycosylated in the cytoplasm with mono- or di-hexoses at 31 sites. Recognition of HMW1 by the receptor on human epithelial cells elucidates their role in pathogenesis. *Streptococcus pyrogenes* responsible for acute pharyngitis and various skin diseases interact with glycosaminoglycans for attachment to the host cells.^24^ Numerous instances, however, have also been accounted for the evolution of host lectins to start over the immune response to bacterial glycans. Therefore, pathogenic bacteria need to produce structures that allow them to hold fast as well as to enter the cell without activating the lectin response to bacteria.

Glycointeraction-mediated immune response evasion or inhibition of host immune functions by expression of glycans on several proteins and surface molecules maximize the probability of continuous growth and transmission to new hosts. Mimicry of host glycan structures by capsular polysaccharides. Group A streptococcus enunciates capsules constituting hyaluronan (the most abundant glycosaminoglycan in the skin), which determine the survival of bacteria on the skin as it interacts with cell surface receptor CD44. Group B streptococcus expresses immunoglobulin-like lectins known as siglecs that bind sialic acids and play roles in both the innate and adaptive immune systems.^25^ Flagellin glycosylation in several bacteria including *B. cenocepacia*, *C. jejuni*, and *Pseudomonas aeruginosa* activates the TLR5-mediated inflammatory response of epithelial cells, which infers a crucial immune evasion stratagem of infecting bacteria. *N*-linked as well as *O*-linked glycosylation systems are recognized in bacterial pathogens. However, *O*-linked pathways are more broadly distributed. Alterations in host glycoconjugates by utilizing scope of glycosyltransferases and glycosidases make the bacteria adopt host's functions ranging from accessing glycans as carbon sources to modify host glycans to foster cell adhesion.

Bacterial toxins target host glycans. AB5 class toxins (subtilase cytotoxin) from *Vibrio cholerae* (cholera toxin), *Bordetella pertussis* (pertussis toxin), *Shigella dysenteriae* and Shiga toxigenic *E. coli* (STEC) (Shiga toxin) and neurotoxins from *Clostridium botulinum* (botulinum toxin) and *Clostridium tetani* (tetanus toxin) are a few best-studied toxins. Apart from the aforementioned widely studied toxins, the interaction with the glycan receptor and the cytotoxicity produced by several bacterial toxins has been studied recently. A toxin from *Salmonella typhii* (typhoid toxin) was reported to have a unique A2B5 architecture comprising a cytolethal distending toxin subunit B homologue (CdtB) and a pertussis-like toxin subunit A (PltA) linked to a homopentameric crown of PltB receptor-binding subunits and selective targeting of the glycans terminating in sialic acid *N*-acetylneuraminic acid (Neu5Ac) over those terminating in *N*-glycolylneuraminic acid (Neu5Gc).^26^ Pneumolysin, a cholesterol-dependent cytolysin isolated from *S. pneumoniae*, is dependent on cholesterol for its cytolytic activity.

### Fungal pathogens

The fungal kingdom bestows a tremendous diversity of glycan structures that comprehend the cellular walls, molecules for energy storage and elaborate surface structures. The majority of fungal cell wall proteins constitute highly mannosylated *N*- and *O*-linked glycans and the glycosylphosphatidylinositol anchor that assists in adjusting the growth conditions in a dynamic and adaptable manner and proffer high mechanical soundness. Glycosylphosphatidylinositol present in the cell membrane performs a momentous role in the synthesis of the cell wall and carries various cell surface proteins to accomplish post-translational modifications that enhance the morphology of the cell.^27,28^ Glycosphingolipids (the major glycolipids in fungi) are required by fungal species for maintaining the fluidity and permeability of the cell membrane. *Candida albicans*, the predominant cause for major infections ranging from oropharyngeal candidiasis, vulvovaginal candidiasis, and invasive candidiasis, contains abundant *O*-glycans. These structures play a major role in the pathogen's interactions with host cells, including macrophages and dendritic cells. *N*-glycans of *Aspergillus fumigatus* comprise mannose structures with the absence of extensive elaborations, whereas *O*-glycans embrace the terminal glucose and galactofuranose alterations.^29^ The cryptococcal capsular structure is a complex mannan substituted with xylosyl and glucuronyl units, namely, glucuronoxylomannan. Moreover, galactans, mannoproteins and chitin-derived molecules associated with the capsule are widely studied for immunological and pathological mechanisms.^30^ Sialic acids observed at the terminal glycan structures decorating the surfaces of cells are nine-carbon structures derived from neuraminic acid. The expression of sialic acids in several pathogenic fungi, for instance, *Candida albicans*, *Cryptococcus neoformans*, *Aspergillus fumigatus*, *Paracoccidioides brasiliensis*, *Sporothrix schenckii*, and *Fonsecaea pedrosoi* have suggested interactions with the host. However, the apparent role of sialic acid remains an unexplored field in fungal biology.

Fungal lectins gained prominent interest in the past decade because of their intriguing sugar specificities, and their biological activities ascend to a wide range of potential pharmacological and biotechnological implications.^31,32^ Discovered lectins have been reported to manifest broad specificity towards varied carbohydrates and glycoproteins. The main characteristics of these lectin domains are that the majority are glycoproteins. Fungal lectins are involved in several cell–cell interactions, mitogenesis, antiproliferation, apoptosis as well as immunomodulation of immune cells.^33^ In contrast to the late apoptotic events in bacteria employing lectin-glycan interaction, fungal pathogens have been reported to induce early apoptosis and inactivation of anti-apoptotic proteins released by host cells. Fungal lectins have been reported to bind to a number of receptors in human host cells. Among the several glycosylated proteins, a major class of pattern recognition receptors including galectin-3 receptor/dectins and toll-like receptors sense and recognize the fungal lectins. Toll-like receptors play a leading role in the innate immune response against the pathogenic fungal species. Of the many lectin interacting receptors, an increase in the activation of toll-like receptors 2 and 4 is generally reported in the event of fungal invasion. The increase in the expression of dectin upon administration of fungal species further aids in the identification of various pathogens.

### Viral pathogens

Viral surfaces and surfaces of host cells are decorated with complex glycans that assume multifaceted roles. The structural complexity and auxiliary heterogeneity of the glycans arise primarily from the utilization of a non-template-driven host cell biosynthetic machinery incorporating enzymes that demonstrate tissue-specific sensitivity.^34^ The glycans on the surface of viral particles are posttranslationally modified enveloped proteins as observed in flaviviruses (Zika virus, hepatitis C, dengue virus, *etc.*) or glycoprotein spike (influenza A, hepatitis B, Lassa virus, Ebola virus, coronavirus, Nipah virus, *etc.*) or viral fibers as in adenoviruses and so on. The complex glycans not only provide stability for proteins and viral particles as a whole but also play a key role in the host immune response to counter the viral infection. The surface glycans can be categorized into high mannose type, acidic (sialylation at terminals) and neutral (uncapped with sialic acid blockade). The interaction of viral surface glycans with the glycoproteins on the host cells either help in intruding the target cell or help the host with the recognition of viral glycans by several circulating glycoproteins that mediate clearance of the virus or by anchored glycoproteins on antigen-presenting cells that prime host immune response to target the viruses.^35^ Acidic glycans on the host surface attract viral particles based on the negative charge and mediate viral entry. Specifically, the heavily sulfated sugar residues (heparan sulphate) are known to be involved in co-receptor-mediated attachment of viruses including the Ebola virus, HIV, coxsackievirus A9 and B3 strains, enterovirus EV71, dengue virus, and echovirus. Sialylated glycan receptors mediate the infection more directly. Hemaglutinin antigen of the influenza virus (the most common, deadly virus) binds to the sialylated glycan receptors on the host surface expressed in the upper respiratory epithelia of humans and leads to transduction of endocytosis signals in host cells. Additionally, the cleavage of sialic acid from both the host cellular surface and the viral surface by an enzyme proffers enhancement of infection productivity and prevention of the self-aggregation of influenza virions emerging from productive infection in a host cell. Similarly, neutral glycans present in bodily fluids and epithelia of the human intestine act as viral entry-attachment pathways for a majority of norovirus genotypes.^36^ Host glycan receptors responsible for the entry (the major virulence factors) of few common viruses are discussed in Table 1.

**Table 1:** Virus recognition of glycan receptors

S. no.	Family of virus	Virus	Type	Host glycan receptors
1	Orthomyxoviruses	Influenza A	Negative sense RNA virus	Sialic acid containing glycan
2	Orthomyxoviruses	Avian virus	Negative sense RNA virus	NeuAca2-6Gal linkages (human-type receptor specificity), NeuAca2-3Gal (avian-type receptor specificity)
3	Orthomyxoviruses	Influenza B	Negative sense RNA virus	9-*O*-Ac-NeuAc
4	Orthomyxoviruses	Isavirus	Negative-sense single-stranded RNA	4-*O*-Ac-NeuAc
5	Paramyxoviridae	Parainfluenza virus	Negative-sense single-stranded RNA	NeuAca2-3Galb1-4GlcNAc
6	Paramyxoviridae	Sendai virus	Negative-sense single-stranded RNA	Neu5Aca2- 3Galb1-3GalNAc and O-linked glycans terminal sequences
7	Coronaviridae	Alpha-coronavirus	Positive-sense RNA viruses	NeuAc or NeuGc as secondary receptors
8	Coronaviridae	Beta-coronavirus and torovirinae subfamily	Positive-sense RNA viruses	9-*O*-AcNeuAc, 4-*O*-Ac-NeuAc, or other O-Ac-SAs
9	Coronaviridae	MERS	Positive-sense RNA viruses	NeuAc2-3Gal containing glycans as secondary receptor
10	Picornaviridae	Enterovirus	Single strand, positive-sense RNA viruses	NeuAca2-6Gal and/or NeuAca2-3Gal linkages
11	Arenaviridae	Lassa fever virus (LFV) and lymphocytic choriomeningitis virus (LCMV)	Bi-segmented negative-sense RNA viruses	a-dystroglycan
12	Polyomaviridae	Mouse polyomavirus	Isohedral, double stranded DNA	NeuAca2-3Galb1-3GalNAc and O-linked glycoproteins
13	Polyomaviridae	Human BK and JC polyomaviruses	Isohedral, double stranded DNA	NeuAca2-3Gal and NeuAca2-6Gal sequences
14	Parvoviridae	Protoparvovirus and, feline panleukopenia virus	Isohedral, single stranded DNA	NeuGca2-3Gal terminated sialosides
15	Parvoviridae	Minute virus of mice	Isohedral, single stranded DNA	NeuAca2-3Galb1-4 (Fuca1-3)GlcNAc and NeuAca2-8a2-8NeuAca2-3(8)Gal (NeuAc)
16	Parvoviridae	Adeno-associated virus-9	Isohedral, single stranded DNA	Galactose and unknown *N*-glycan
17	Parvoviridae	Parvovirus B19	Isohedral, single stranded DNA	Globoside glycolipids
18	Reoviridae	Animal strains	Isohedral, double stranded RNA virus	NeuAca2-3Gal/GalNAc sequences
19	Reoviridae	Human strains	Isohedral, double stranded RNA virus	Sialic acid
20	Caliciviridae	Murine norovirus	Single stranded positive sense RNA virus	Sialic acids on O-glycans
21	Caliciviridae	Feline calicivirus	Single stranded positive sense RNA virus	Sialic acids on *N*-glycans
22	Adenoviridae	HAdV-D	Isohedral, double stranded DNA virus	NeuAca2-3Gal terminated glycans
23	Adenoviridae	HAdV-52	Isohedral, double stranded DNA virus	NeuAca2-8NeuAca2-8
24	Adenoviridae	Animal AdVs	Isohedral, double stranded DNA virus	NeuAca2-3- and NeuAca2-6-specific and LacNAc

A number of viruses employ an important scheme to misguide the humoral immune response by secretion/shedding of glycoproteins. For instance, the glycoprotein gene of Ebola virus constituting a dimeric secreted glycoprotein (sGP) assists in the immune evasion by acting as an antibody decoy by redirecting the humoral immune response towards target epitopes shared by full-length epitopes. The sGP mimics the glycan component of the glycoprotein to direct antibodies towards sGP rather than towards glycoprotein.^37^ Non-structural protein-1 (NS-1) secreted by dengue virus-infected cells subverts the immune system. The *N*-linked glycosylation sites of NS-1 accountable for the replication of the viral genome, facilitating protein secretion and stability, are hypothesized to facilitate vascular leakage upon its immune recognition on endothelial cells.^38^ Furthermore, the glycosylation of envelope proteins to protect the protein surface has been observed in several viral species to evade the host immune system. Ebola virus glycoprotein is involved in the steric shielding of several cell membrane ligands to immune receptors including MHC class-I polypeptide-related sequence A and human leukocyte antigen class-I. The two highly glycosylated domains of the glycoprotein-mucin-like domain and the glycan cap domain are reported to impair the immune function. The role of *N*-glycans and their contribution to the phenomenon of immune evasion were reported.^39^ The significance of *N*-linked glycosylation of envelope proteins, regarding immune evasion, has been observed across many viruses including hepatitis C, hepatitis B, Hendra, Nipah, influenza, HIV, Newcastle disease and herpes simplex virus. The role of glycosylation of newly emerged SARS-CoV-2 spike protein in immune evasion by camouflaging immunogenic protein epitopes is being validated.^40^

The presence of self-glycans, though poorly immunogenic, aids in triggering the host immune response. Glycans of glycoproteins are recognized by the host antibody response. Moreover, mammalian lectins, for instance, the calcium-dependent lectins involving the dendritic cell-specific intercellular adhesion molecule-3 grabbing non-integrin (DC-SIGN), macrophage mannose receptor (MMR), mannose-binding lectin (MBL), and soluble lectin surfactant-associated proteins A and D (SP-A and SP-D) participate to both impede and facilitate viral propagation. Similarly, the binding of galectin-1 to β-galactoside-containing sugars escalates the infection of target cells such as macrophages to promote HIV infection and CD4+ T cells to promote HIV and human T-lymphotropic virus infections but impede attachment of Nipah virus to its target cells.^41,42^

## Hunting the cancer itinerary with ingenuity

**Fig. 2:**
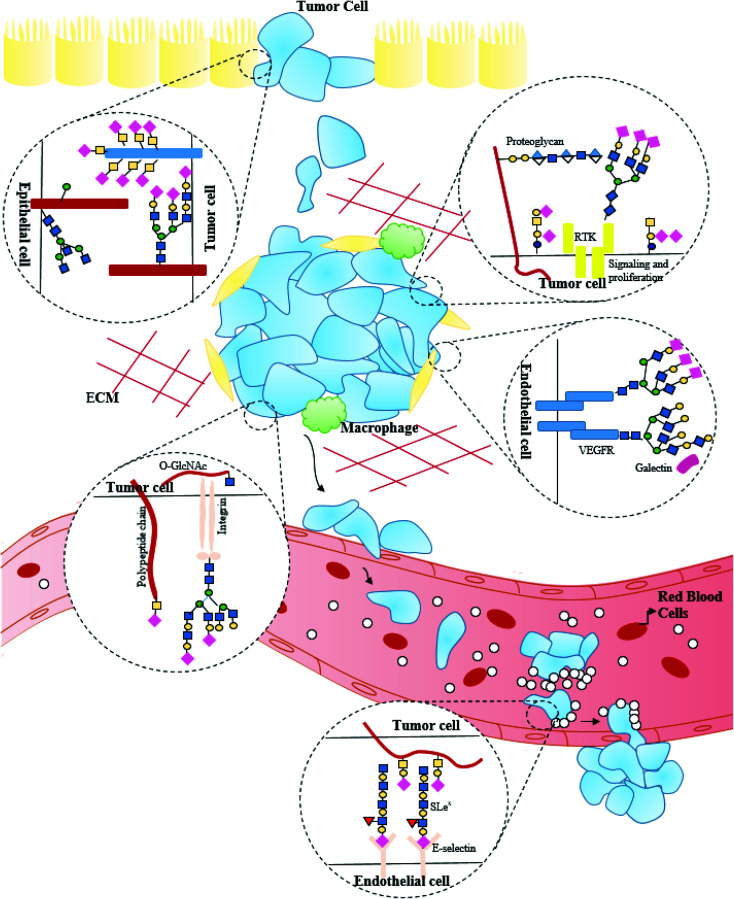
Glycans aid in cancer development and progression.

Cancer growth is dependent on the capability of tumor cells to go past the cellular cycle checkpoints and evade the signals inducing apoptosis, immune reconnaissance and migration to metastatic sites. Glycosylation patterns are a hallmark of cancer phenotypes. The expanding sialyl Lewis structures, increased branched structures of *N*-glycan or revelation of mucin-type *O*-glycan, abnormal addition of fucose sugar units (core fucosylation), Tn antigen, *etc.*, amount to the alterations in the glycosylation pattern observed in cancerous cells. Glycan composition pattern evolves with the onset of the disease and changes in parallel to the cellular metabolism, as the infection grows and takes over the body.^5,43^ An increase in the fucosylation levels coupled with increasing levels of fucosylated glycoforms observed in the fifth most common cancer-hepatocellular carcinoma can be utilized for early detection of the disease.^44^ The significance of sialylation and sialylated glycans in breast cancer brain metastasis was studied.^45^ Changes in glycosylation emerge due to the alteration in expression levels of glycosyltransferases in the Golgi compartment of tumor cells. Loss of the polypeptide *N*-acetylgalactosaminyltransferase 3, for instance, can prompt a significant increase in the pro-growth and post-metastatic antigen produce (for example, Tn and T antigens on receptor tyrosine-protein kinase erbB2) and repression of enzyme Gal 3(4)-l-fucosyltransferase can inhibit metastasis and suppress growth by forestalling Lewis Y antigen (LeY) production on receptor tyrosine-protein kinase erbB2.^46,47^ The role of mucin-type *O*-glycans in the proliferation of tumors (overexpression of MUC4 on mammary tumors, malignant melanoma, ovarian tumor, pancreatic tumor, *etc.*, and its absence in normal cells) in inducing rapid cell growth, suppression of tumor, *etc.*, have been widely studied. MUC4 gene interacts with ERBB2 (frequently overexpressed in cancerous cells) in place of ERBB ligands. The contribution of *N*-glycans in protein-folding, half-life and quality control, cell–cell recognition, adhesion and signaling, however, makes it hard to harmonize the cancer growth with alteration in glycosylation at specific proteins. Howbeit, *N*-glycosylation inhibitors have been reported to inhibit the survival of several tumors.^48^ The proliferation of cancerous growth is reliant on the heparan-sulfate proteoglycans. Tumor cells of pancreas, breast, ovary, hepatocellular cancers, *etc.*, exert modifying influence on cell surface heparin sulfate proteoglycans in a way to enhance their binding capacity for growth factors and activation of receptor tyrosine kinases. Integrin, a heterodimer glycoprotein constituting α and β subunits, has received considerations concerning their carcinogenic procedure and malignant progression. *N*-glycosylation on integrins indirectly binds with the receptor glycoproteins, which could be mediated by some lectins and glycosphingolipids. The role of glycosylation in cancer growth and proliferation is illustrated in Fig. 2.

Glycosylation of distinct tumor tissues and proteins in serum aid in the ambiguous identification of the disease, patient prognosis, and responses to the undergoing therapy. Glycosylated protein markers including the prostate-specific antigen (observed to alter in prostate cancer), carcinoembryonic antigen (observed to alter in bladder, colorectal, pancreatic, breast, and lung cancer), haptoglobin, transferrin, and hemoglobulin have been reported. Glycans modulate several facets of the immune response that can meddle with the anticancer reaction of the immune framework, prompting the development of malignant growth cells impervious to the immune framework of the body. Lectins binding glycans help tumors abscond immune surveillance. The discovery of diagnostic glycoprotein markers by the trapped glycoproteins utilizing lectins or other glycan-binding antibodies has proffered the detection of glycosylation between cancer and healthy controls.^46^ MUC1, MUC16, carcinoembryonic antigen (CEA), and prostate-specific antigen (PSA) are some of the examples of glycoprotein diagnostic biomarkers. Selectins expressed by lymphocytes, platelets, and endothelial cells bind to sialofucosylated moieties and play a pivotal role in homing and tissue recruitment of leukocytes. The expression of sialofucosylated antigens, including SLe^x^ and SLe^a^, has been shown to facilitate the arrest of circulating tumor cells on endothelial cells and is strongly associated with increased metastatic potential. Sialyl Lewis A (sLe^a^) carbohydrate motif recognized by the specific mouse monoclonal antibody on a monosialoganglioside was initially identified in gastrointestinal cancer, which is now identified in different tumors including gastric, pancreatic, and colorectal cancers, underlining anomalous glycosylation as a principal highlight of malignant growth pathobiology. Glycans from proteins are cleaved utilizing enzymes or specific chemicals to recognize the biomarkers from released glycans. *N*-linked glycans are cleaved utilizing peptide-*N*-glycosidase, whereas alkaline sodium borohydride is majorly involved in cleaving *O*-linked glycans. Both *N*- and *O*-glycans can be cleaved simultaneously using hydrazinolysis. Glycan enrichment by solid-phase extraction is then followed by compositional and structural analysis. Isolation of attainable targets from biological mixtures proffers protein-specific biomarker information.^49^ Although the utilization of a solitary biomarker for the recognition and monitoring of the advancement of an ailment is an alluring possibility, entire glycome profiles may be superior to a solitary glycosylation design for the evaluation of cancer progression.

Chronic cell proliferation is regulated by the degree of glycosylation of growth factors including epidermal growth factors, fibroblast growth factor receptors, platelet-derived growth factors, and insulin-like growth factors. Howbeit, in addition to the induction and maintenance of positively acting growth simulatory signals, cancerous cells are required to evade the growth suppressors, including p53 and retinoblastoma proteins. *O*-GlcNAcylation of suppressors could amplify the pro-oncogenic activity by inhibiting phosphorylation. Moreover, the modification of glycans on cell death receptors including Fas (CD95) and TNFR1 (tumor necrosis factor receptor 1) enable the tumor cells to resist apoptosis. Besides, the key element of cancerous cells is aerobic phosphorylation. The increased uptake of glutamine and glucose to adapt to the increased energetic and biosynthetic needs of the tumor results in a shift of metabolism from oxidative phosphorylation. The elevated levels of *O*-GLcNAc in both *N*-glycosylation and *O*-glycosylation portray the sign of malignant growth by acting as nutritional sensors.^50^

## Analytics for study of therapeutic activity of the complex

The pronounced increment in the delineation of analytical techniques effectively appertained to the investigation of complex glycans and glycoconjugates, involving mass spectrometry, high-performance liquid chromatography, lectin-based array, isoelectric focusing and capillary electrophoresis, which have helped decipher historically unmet clinical challenges in several disease areas.^51^ Legion of these advanced technologies have unambiguous precedence in contrast to customary techniques, comprehending capability to examine minute measures of biologically based materials. Furthermore, because of the high resolving power, these state-of-the-art techniques proffer the ability to separate between closely related glycan structures. To deal with the intricacy of structure and diversity of complex glycans, hyphenated strategies—analysis based techniques integrating parts of segregation, systematic estimations and, in certain occasions, coordination of data through bioinformatics—are indispensable for the characterization of glycan structures. Analysts have endeavored for the development of chip-based separation and analytics; strategies including fluorescent labeling of ligands and high-performance liquid chromatography coupled to imaging analysis, and combining chip-based separation with mass spectroscopic analysis, electrospray ionization-time of flight with mass spectroscopy, capillary electrophoresis with mass spectroscopy and matrix-assisted laser desorption ionization with mass spectroscopic analysis have been reported.^2,51^ A novel screening method designed combining electrospray ionization with mass spectroscopy was employed for the comprehensive mapping of glycan interactions. The quantitative high-throughput strategy screened *Glycan libraries* against glycan-binding and glycan-processing proteins.^52^

Lectin microarrays have emerged as propitious analytical tools for glycan profiling. Lectins with known specificity are immobilized as microdots on a solid surface. The interaction of the binding glycans with the corresponding lectins enables expeditious discernment of their specificity and record of even very weak interactions with high specificity.^53^ A standardized technique for glycome profiling of whole body under different physiopathological states was reported. The lectin-assisted tissue glycome mapping was performed on both *N*-and *O*-glycome profiles of various regions within tissue sections of the brain, liver, kidney, spleen, and testis of two C57BL/6J mice.^54^ Lectin affinity chromatography is employed as a common method for isolation, fractionation, and purification of carbohydrate-containing structures. The non-covalent character of lectin-carbohydrate interactions is characterizable by a frankly low affinity, thus allowing glycans to be competitively displaced from the specific complex by a competitor compound and hence proffering additional opportunities in the implementation of lectin affinity chromatography. Lectin-based affinity techniques as effective tools for targeting, separation, and reliable identification of glycans have recently been utilized as biomarkers for breast and prostrate cancers.^55,56^

## What is in the medicine cabinet?

Progress in the field of glycobiology has facilitated the development of glycan-based therapeutics. Glycoprotein therapeutics balance protein action, stability, pharmacokinetics, pharmacodynamics, bioactivity, and safety. Biopharmaceuticals including monoclonal antibodies, antibody–drug conjugates, and other recombinant protein products (for instance, fusion proteins, growth factors, cytokines, therapeutic enzymes, and hormones) have been developed against cancer, autoimmune diseases, and other potentially fatal diseases.^2,57^

Several therapeutic approaches have been investigated concerning the potential of glycosylation engineering. Advances in metabolic glycoengineering, involving the exploitation of the substrate promiscuity of specific glycosylation pathways to integrate the non-natural monosaccharides into cellular glycans, have transmogrified the far-reaching procedure to manipulate the glycosylation in living cells to progress in health care on multiple fronts. The processing of altered glycoforms constituting non-natural chemical functionalities proffers a robust methodology to alter the cellular functions and responses.^1,58^ Safety, viability, and serum half-life of immunotherapeutics including immune cells and associated enzymes, antibodies, hormones, and cytokines are controlled by regulation of glycosylation. Supplement of tagatose and sucrose in host cell culture media proffers a persuasive methodology for the targeted modulation of protein glycosylation profiles in cultured cell lines. Supplementation of host cell culture media effectively tweaks the *N*-glycan glycoform profile towards high mannose species and diminishes the overall degree of fucosylation for guaranteeing biologic comparability for recombinant glycoprotein therapeutics.^59^ Metabolic glycoengineering procedures have emerged extraordinarily worthy of treatment of cancer. Nutrient-deprived cancerous cells hastily and robustly incorporate the exogenously supplied sialic acids. Pertinent supplement of sialic acid conjugates, as well as nutrient-deprived conditions, extends the exploitation of an expanding miscellany of functional groups including alkenes, ketones, and “*N*-glycolyl” hydroxyl groups, and additional carbon atom modification to *N*-acyl groups, azide groups, and photo-activated functional groups such as phenyl azides or thiol and diazarines groups to selectively tag “glycoengineered” sialic acids has prodded enthusiasm for utilizing these strategies for tumor analysis/diagnosis or treatment.^60^ Sodium butyrate applied in the cell culture media enhanced the protein expression in Chinese hamster ovary cell lines expressing human erythropoietin or immunoglobulin G. Butyrated alternatives to *N*-acetylmannos-amine (an intermediate metabolite in the sialic acid pathway) increased the sialic acid production to much lower concentrations in contrast to the natural *N*-acetylmannos-amine, thus providing a propitious chemical analog to conventional chemical treatment strategies as a means for therapeutic protein production.^61^ Delivery of 1,3,4-*O*-acetyl-*N*-azidoacetylmannosamine to folate receptor overexpressing cells that bind to the antibody-recruiting molecules serves as an alternative approach to customary chemotherapeutics in cancer therapy and other potential diseases. Conversion of Ac3ManNAz to cell surface glycans bearing azido functionality serves as an anchor for the introduction of l-rhamnose as a hapten, a moiety responsible for the initiation of the immune response as mark targets as “permit to kill” by complement-dependent cytotoxicity and antibody-dependent cellular phagocytosis.^62^ Metabolic engineering of bacterial glycans further stands promising because of high tissue permeability endowed by small molecules and the low probability of disrupting bacterial populations.^63^ Metabolic engineering and unnatural monosaccharides utilized to globally inhibit the bacterial glycome by focusing on a small panel of metabolic inhibitors have paved the way for the development of a broad array of metabolic inhibitors to study and alter bacterial glycans in diverse species.^64^

Lectin has seen diverse applications in biological research and pharmacology. Given the lectins' potentiality to explicitly perceive cell surface sugars, it can presumably concede specificity to medication conveyance frameworks or to help in diagnosis. Germane to the discovered drugs targeting human lectins, Uproleselan and Rivipansel are in the late-stage trial of acute myeloid leukemia and sickle cell anemia respectively. Interestingly, the galactose-specific lectin activity of the multi-drug-resistant *Neisseria gonorrhoeae* has been recognized lately and peptide mimicry of the galactose-binding region has been investigated for the novel-host-targeted therapeutics for multi-drug-resistant gonococcal infection in women.^65^ Moreover, lectins are utilized as drug carriers to target glycans on the cancer cell surface. rBC2LC-N lectin–drug conjugate discovered by the fusion of selected toxins with bacterial exotoxin has exhibited prominent cytocidal effects against pancreatic ductal adenocarcinoma.^66^ The therapeutic potential of lectibody Avaren-Fc (AvFc), a high mannose glycan-binding lectin-Fc fusion protein, suggests a safe and efficacious means to prevent the hepatitis C viral infection upon liver transplantation in end-stage liver disease patients. The lectibody binds to clusters of high-mannose glycans on the envelope protein of viral particles.^67^ Significant therapeutic targets for anti-adhesive treatment and compounds aimed at emulating the human glycoconjugates have been developed. Lectins such as DCSIGN, siglecs and galectins, with significant functions in inflammation or immune system activation, have been appraised as the principal targets. Sialic acid sugars, working as ligands of the immunosuppressive sialic acid-binding immunoglobulin-like (Siglec) lectins, are becoming prominent as key regulators of the immune system. Aberrant sialic acid–siglec interactions have been related to several diseases including autoimmunity, infection, inflammation, aging or cancer.

Marine-sourced glycans in recent decades have made marvelous contributions in the growth of promising therapeutics. Carrageenan and agarans, the two types of sulfated glycans and their derivatives extracted from red algae, have been extensively investigated for their manifold biological activities including anticoagulation, antiviral effects, cholesterol-lowering effects, immunomodulatory activity, and antioxidation. Heparin is the oldest carbohydrate-based drug in the market. What is more, it is one of the most recommended drugs depicted today as an anticoagulant and antithrombotic agent. Marine-derived heparinoids are structurally comparable to heparins, and they exhibit biological properties similar to glycosaminoglycans including but not limited to sulfated alginates, dextran sulfate, and carrageenans.^4,64^

Presently, the thriving sugar-based drugs are apparently the antiviral compounds Tamiflu and Relenza. The neuraminidase ligands are apt in blocking the binding site to prevent the viral release, cell-to-cell spread, and reduction of magnitude and duration of symptoms of the H1N1 virus and the influenza virus respectively. Concerning several common viral pathogen infections, highly sulfated synthetic glycomimetics are reported inhibitors of viral binding infection. Glycomimetics involve the imitation of structural and functional aspects of glycans to exploit the chemical information encoded by the glycans by controlling and altering the information they direct. A novel class of carbohydrate-derived products has drawn significant attention as not just the spatial and the geometric properties are retained but also the biological properties are modulated. The clinical trials of iota-carrageenan and Carraguard (PC-515) treatment of H1N1 and HIV respectively are presently being studied to define the therapeutic index of the aforementioned drugs in detail.^68^ Sulfated glycomimetic oligomers and polymers have also been reported to proffer prospective broad-spectrum antiviral activities against Influenza A virus, human papillomavirus, merckel cell polyomavirus and herpes simplex virus.^69^ Synthetic glycopeptides mimicking natural HIV envelope glycoproteins have also been envisaged as anti-HIV therapy. Viral envelope glycoproteins at the surface of several viruses are targets for virus neutralization. These proteins, such as gp120 in HIV-1, are decorated with high-mannose-type glycans, which are the first structures to be encountered by the host immune system. While these *N*-glycans are recognized as self-antigens and help to evade neutralization, much more potent antigenic responses can be obtained targeting both internal glycans and the protein surface. Hence, mimicking glycopeptides can be envisaged as a therapeutic strategy to elicit a broad antibody response^70,71^

Bacterial glycans represent intriguing targets of therapeutics and diagnostics. Carbohydrate epitope-dependent bacterial vaccines are at the vanguard of prophylactics and are utilized to immunize children against bacterial pathogens including *Haemophilus influenzae*, *Streptococcus pneumonia*, and *Neisseria meningitides*.^72^ However, antibiotics are crucial for individuals suffering from active bacterial infection. Indeed, our antibiotic arsenal contains small molecule inhibitors of enzymes involved in bacterial glycoconjugate biosynthesis. Among the most prominent of these are the blockbusters vancomycin, bacitracin, and penicillin, all of which interfere with peptidoglycan biosynthesis. Although these antibiotics have saved countless lives, the emanation of antibiotic resistance has instigated search for alternative treatments. Multimeric boronic acids that specifically attack the cellular surface glycans kill mycobacteria and exhibit low cytotoxicity to a panel of human cells, thus providing a pathway for the development of a next-generation anti-tubercular therapy.^73^ The intracellular survivability of several pathogenic bacteria after the intrusion of host eukaryotic cells is pivotal as the intracellular niche spares protections from diverse aspects of the host's immunity. Provided that the primary function of innate immune cells is to destroy pathogens, the survival of intracellular pathogens in the cytosol remains a paradox. Prodrugs have emerged as a popular and widely accepted strategy to enhance the biopharmaceutical, physiochemical, and pharmacokinetic characteristics of propitious drug candidates, for instance, ciprofloxacin and norfloxacin. In a recent paper, the synthesis of glycan-targeted polymeric antibiotic drugs to combat intracellular pathogens in the alveolar macrophage has been reported.^74^

Glycan-based precision medicine diagnostics are at an early stage. Precision medicine involves the prevention and treatment of diseases by taking into consideration the variability in genes, environmental conditions, and stages of the disease and nutritional needs of an individual. Glycans are vital regulators of several bioprocesses that influence person–person variability in their propensity to respond to common complex diseases and drug exposure.^75^ Glycomics can essentially supplement the genomics and proteomics tools that are now being assessed for creating customized medications. However, the primordial challenges including the requirement of experimental procedures for the authentic and reproducible measure of the structure or sequence of complex glycans and a system for delineating the sequences with respect to their properties that lead them to their functions still need to be overcome to realize the vast potential of glycans.

## Experience nano

Exploration and exploitation of the unique properties of the glycan structure require a closer setting in contrast to the cellular landscape. Nanotechnology moves from the angstrom to the nanometer range (from ∼10^−10^ to ∼10^−7^ m) to create, manipulate, and characterize structures on those scales. Nanotechnology has been applied to glycobiology forming the new science of glyconanotechnology. Glyconanomaterials profit from the unique chemical and physical characteristics of nanoscale including photonic, magnetic, electronic, or catalytic characteristics that seem to be invisible in the bulk together with the characteristics of glycans including structural diversity, biocompatibility, and water solubility targeting properties.^76^ Glyconanotechnology aids the development of biosensors and strategies for screening lectins, glycans, pathogens or cancerous cells. Nanoengineered glycan sensors may also aid in the glycoprotein profiling without labeling or glycan liberation steps.

Glycan arrays are a significant step in the path towards more sophisticated carbohydrate nanotechnologies because they shed light on glycan-binding affinity and selectivity. Glycan arrays incorporate solid surfaces that portray a library of glycans spatially encoded into micrometer-scale spots, and the resulting surfaces are utilized to expeditiously and combinatorially evaluate the affinity and selectivity of glycan-binding proteins to the immobilized glycans. Improvement of surface chemistry to acquire a coordinated, multivalent binding interaction and growth in the glycan library within the arrays is restricted by the complexity of preparing carbohydrates or procuring natural samples of glycans. The immobilization chemistry—the chemical associations and the interactions that bind the glycans onto the substrates—majorly affects the performance of the glycan array. Binding of glycans utilizing the supramolecular interaction including ionic, Van der Waals, solvophobic, and hydrogen bonding interactions constitute the noncovalent printing strategies. The approach witnesses several advantages including reduction in the number of synthetic steps for glycan printing, easy fabrication of the surfaces, and high success of attachment. Howbeit, covalent printing involves the formation of covalent bond between the glycan and the surface with the potential to control glycan orientation and density by accessing the selectivity of organic chemistry.^77^ By contemplating ideas from polymer science, specialists have as of late made progress that disentangle the arrangement of complex glycan chips, enhance library assorted variety, and explain the subtle interplay between carbohydrate density and binding affinity. In polymer-based methodologies, the foundation of either a normally inferred biopolymer or a manufactured polymer is utilized as a platform to set up a multivalent saccharide display that extends away from the substrate, and in doing so make a coupling domain that intently resembles the cellular surface, and diversity can be increased by varying the spacing, chain length, and carbohydrate presentation.

Glycan conjugated gold nanoparticles are endowed with high aqueous solubility/dispersibility and biocompatibility that form the basis for novel techniques in bioanalytical implications. Gold glyconanoparticles have aided in the clarification of mechanistic aspects of multivalent carbohydrate recognition and are utilized as antiadhesives for the prevention of melanoma metastasis, in molecular as well as cellular imaging and as vaccine candidates.^78^ Glycan-functionalized gold nanoparticles have been applied in the establishment of suitable, portable sensors for clinical diagnostic processes and surveillance of influenza viruses. The potential of nanoprobes to discriminate between fourteen viral strains based on strain, subtype, and origin (human *vs.* avian) levels represents an innovative strategy.^79^ Besides, glycan-decorated gold nanoparticles have been interestingly utilized in surface-enhanced Raman spectroscopy for highly specific detection of Galectin-9.^80^ The ubiquitous nature of carbohydrate–lectin interactions in infectious diseases presents new opportunities in drug design and biosensing. Polymer-stabilised, glycosylated gold nanoparticle platforms allow for high-throughput and importantly label-free screening of carbohydrate–lectin interactions. Identification of lectins with similar carbohydrate-binding preferences removes the need for complex, expensive and inaccessible branched glycans, or antibodies, thereby allowing use in low-resource settings for the identification of pathogens such as cholera.^81^ Thionine-bridged multiwalled carbon nanotubes/gold nanoparticles have been employed in the development of electrochemical biosensors for glycan assays on living cancer cells. Highly sensitive and specific detection of mannose in the human liver and prostate cancer cells paved the path for the analysis of other glycans on living cells with the selection of more lectins.^82^ Magnetic nanoparticles such as manganese and iron oxide nanoparticles have the potentiality to load several ligand copies, and therefore, they are considered better for receptor targeting and multimodal imaging power. Antibodies utilized in drug discovery exhibit superlative specificity but encounter thermal instability, shorter lifetime, and a high cost. Detection of early-stage diseases by successfully mimicking leukocyte recruitment during inflammation and display of multiple copies of oligosaccharides increasing potential multivalency of binding interactions proffer quick translation potentials from mammalian models to humans. Coherent utilization of glycan-imprinted magnetic nanoparticle-based systematic evolution of ligands by exponential enrichment for the screening of aptamers against glycoproteins has been reported.^83^ Quantum dots, the luminescent semiconducting nanomaterials made of binary zinc sulfides, selenides, or cadmium, exhibit tunable optical properties dependent on size. Glycan-conjugated quantum dots are utilized in the study of several carbohydrate-related interactions. Carbon nanotubes are coated with glycopolymers to create *in vivo* biological probes. Multivalent sugar ligand-coated graphenes are utilized for agglutination and inhibition of bacterial species, cancer treatment, photothermal therapy and drug delivery.^84^ Drug delivery systems constituting nanoparticle-galactosylated chitosan/graphene oxide/doxorubicin were utilized for efficient loading of cancer cell targeting characteristics.^85^ Chemical vapor-deposited graphene-based glycan arrays have been utilized as potential sensing arrays on a variety of substrates. The platform enabled the monitoring of glycan–lectin interactions by fluorescence and mass spectrometry.^86^ Nanocarriers based on heparin and their derivatives have been appertained to combat cancer *via* targeted, magnetic, photodynamic, and gene therapy.

## Conclusion

In recent years, several contributors have evinced the manipulation of carbohydrates in therapeutical chemistry. Glycans play several pivotal roles in cellular response to environmental stimuli as well as cellular growth and differentiation; distinct variations in their composition are directly associated with several diseases. The commencement of advances in the technology to face the challenges posed by the complexities of glycoconjugates has ameliorated our grasp of the pathological and physiological processes that are controlled by glycans. Such endeavors are additionally encouraged by improvements in research tools and training in glycosciences both of which expedite the progress of glycomedicine, where glycobiology is applied to the development of novel therapies. The preferable integration of glycomics with extensive biomedical research can aid in the discernment of not just how glycans are altered in cancer, immune dysfunction or other diseases, but also why. This step is also crucial for therapeutics. Emphatically, large-scale screens frequently implicate glycan-processing enzymes in several processes and diseases, making it attainable—and even imperative—for biologists to reckon with glycoscience. Glycointeractions play a central role in pathology. Explicating the glycointeractome is a vital step in the interpretation of the host–pathogen interaction, and tactics to wholly elucidate the glycointeractome have developed rapidly in recent years. Glyconanotechnology has large and presently underexploited potentiality to help interpret the elementary procedures of glycan-mediated interactions and signaling by proffering defined and tuneable model systems on a relevant scale. In this regard, glycan-functionalized nano particles and quantum dots accounted for defined systems to examine the glycan-mediated multivalency and have burgeoned as scaffolds to model glycan-dependent biodistribution and to study the glycan-dependent modulation of immune responses. The field is challenged, however, by the complexity and dynamic nature of the glycome and lack of apprehension of how glycoconjugates are expressed topologically and temporally. The interpretation of their functions and higher-order contributions typically necessitate physiological studies of organisms and the recognition of altered pathways of anabolism and catabolism in patients. Thus, the coalescence of studies in single-cell systems may fail to identify or even predict these higher-order functions. Further, for any type of biological system, the degree of understanding can be gauged by its predictive ability, and on this score glycoscience is truly challenged. The miscellany of probable monosaccharide combinations, linkages and modifications entail diverse techniques and methodologies to accomplish selective recognition of biomedically relevant glycans and their derivatives. Furthermore, the requirement of efficient synthetic and high-throughput screening methodologies for the synthesis of complex glycans is another major challenge. An important area that demands additional effort is the development of comprehensive glycobioinformatics resources that can be utilized to exploit glycomic data by linking the glycan structure, and its expression and function. The development of novel glycan-binding entities will continue to be a high priority for the foreseeable future.

## Conflicts of interest

Nothing to declare.

## Supplementary Material
